# ClickArr: a novel, high-throughput assay for evaluating β-arrestin isoform recruitment

**DOI:** 10.3389/fphar.2023.1295518

**Published:** 2023-11-07

**Authors:** Alexander R. French, Yazan J. Meqbil, Richard M. van Rijn

**Affiliations:** ^1^ Department of Medicinal Chemistry and Molecular Pharmacology, Purdue University, West Lafayette, IN, United States; ^2^ Purdue Institute for Integrative Neuroscience, Purdue University, West Lafayette, IN, United States; ^3^ Computational Interdisciplinary Graduate Program, Purdue University, West Lafayette, IN, United States; ^4^ Purdue Institute for Drug Discovery, Purdue University, West Lafayette, IN, United States

**Keywords:** G protein–coupled receptors, biased agonism, delta opioid receptor, high-throughput drug screen, click beetle luciferase, signal transduction, beta-arrestin

## Abstract

**Background:** Modern methods for quantifying signaling bias at G protein–coupled receptors (GPCRs) rely on using a single β-arrestin isoform. However, it is increasingly appreciated that the two β-arrestin isoforms have unique roles, requiring the ability to assess β-arrestin isoform preference. Thus, methods are needed to efficiently screen the recruitment of both β-arrestin isoforms as they compete for a target GPCR in cells.

**Methods:** We used molecular cloning to develop fusion proteins of the δ-opioid receptor (δOR), β-arrestin 1, and β-arrestin 2 to fragments of click beetle green and click beetle red luciferases. In this assay architecture, recruitment of either β-arrestin 1 or 2 to the δOR generates a spectrally distinct bioluminescent signal, allowing us to co-transfect all three constructs into cells prior to agonist challenge.

**Results:** We demonstrate that our new assay, named “ClickArr,” is a live-cell assay that simultaneously reports the recruitment of both β-arrestin isoforms as they compete for interaction with the δOR. We further find that the partial δOR agonist TAN67 has a significant efficacy bias for β-arrestin 2 over β-arrestin 1 when recruitment is normalized to the reference agonist leu-enkephalin. We confirm that ClickArr reports this bias when run either as a high-throughput endpoint or high-throughput kinetic assay, and cross-validate this result using the PathHunter assay, an orthogonal commercial assay for reporting β-arrestin recruitment to the δOR.

**Conclusion:** Our results suggest that agonist:GPCR complexes can have relative β-arrestin isoform bias, a novel signaling bias that may potentially open up a new dimension for drug development.

## 1 Introduction

Biased signaling at G protein–coupled receptors (GPCRs) has been a focal point of drug design in recent years, with the discovery that “biased” agonists can direct GPCR signaling preferentially via particular partners such as G proteins or β-arrestins promising to produce next-generation pharmaceuticals that have established or new therapeutic effects while lacking deleterious “side effects” that result from the activation of undesirable pathways (Smith et al., 2018; Wootten et al., 2018). However, many of these efforts do not account for the distinct and often opposing roles β-arrestin isoforms can play in disease models (Srivastava et al., 2015) such as rheumatoid arthritis (Li et al., 2011) and Parkinson’s disease (Fang et al., 2021), as well as in modulating the effects of psychostimulants (Zurkovsky et al., 2017), psychedelics (Rodriguiz et al., 2021), and opioids (Ko et al., 2021). At the molecular level, the two isoforms play different roles in regulating GPCR internalization and trafficking (Kohout et al., 2001; Pradhan et al., 2016; Wang et al., 2022). These behavioral and functional differences may be driven by changes in β-arrestin 1 and β-arrestin 2 conformations following binding to the phosphorylated C-terminal tails of GPCRs (Min et al., 2020; Haider et al., 2022).

To take full advantage of the therapeutic potential of GPCRs, biased agonists would ideally account for differences in β-arrestin isoform signaling (Srivastava et al., 2015). In cells, β-arrestin 1 and 2, also called arrestin-2 and arrestin-3, respectively, compete for binding to GPCRs. In currently available drug screening platforms, a single isoform is overexpressed and this competition is not accounted for. Moreover, when measured separately, comparing the activity of both isoforms requires normalizing across different tests and even cell lines. Thus, comparing the performance of both β-arrestin isoforms doubles the time and material costs of the study, discouraging it in common practice.

Here, we present ClickArr, a click beetle luciferase–based assay for arrestin recruitment that allows the simultaneous readout of both β-arrestin 1 and 2 from the same cell. ClickArr is optimized to run in multi-well plate format for the fast evaluation of lead compounds and arrestin signaling characteristics. We further apply the ClickArr architecture toward panning agonist signaling at the δ-opioid receptor (δOR), which we demonstrate can show ligand-directed bias toward β-arrestin 2 over β-arrestin 1.

## 2 Materials and methods

### 2.1 Molecular cloning

We created the original δOR fusion to a C-terminal click beetle green (CBG) fragment (CBGct) (Wood et al., 1989) from CBG99 by digesting a pCDNA3.1 vector containing an N-terminally FLAG-tagged mouse (*Mus musculus*) δOR sequence (Evans et al., 1992), generously provided to us by the Whistler Lab, with *NotI* and *PasI*. Into this, we ligated a *NotI*/*PasI*-digested fragment of a custom-ordered vector encoding the sequence /*NotI*/-δOR (res. 338–372)-Gly-Ser-CBGct (res. 395–542)-STOP-/*PasI*/. To generate the original fusion of an N-terminal CBG fragment (CBGnt) from CBG99 to β-arrestin 1, we digested a pCDNA3.1 vector encoding human β-arrestin 1 (cDNA.org) with *NheI*/*BspEI*. Into this, we ligated a *NheI*/*BspEI*-digested fragment of a custom-ordered vector encoding the sequence/*NheI*/-CBGnt (res. 1–413)-Ser-Gly-Leu-Lys-Ser-Arg-Arg-Ala-Leu-Asp-Ser-Ala-β-arrestin 1 (res. 2–169)-/*BspEI*/. Similarly, to generate the original fusion of an N-terminal fragment of click beetle red luciferase (CBRnt) to β-arrestin 2, we digested a pCDNA3.1 vector encoding human β-arrestin 2 (cDNA.org) with *NheI* and *AgeI*. Into this, we ligated a *NheI*/*AgeI*-digested fragment from a custom-ordered vector encoding /*NheI*/-CBRnt (res. 1–413)-Ser-Gly-Leu-Lys-Ser-Arg-Arg-Ala-Leu-Asp-Ser-Ala-β-arrestin 2 (res. 2–197)-/*AgeI*/. For each, the portion of encoded δOR, β-arrestin 1, or β-arrestin 2 was set to replace the original sequence cut out by the restriction site without altering the protein sequence in the final construct. An exception to this were the arrestin constructs where the start codon methionine was removed as it was no longer necessary once the N-terminal luciferase fragments were added. All ligations were performed with T4 DNA Ligase (New England Biolabs) according to the manufacturer’s guidelines.

**FIGURE 1:**
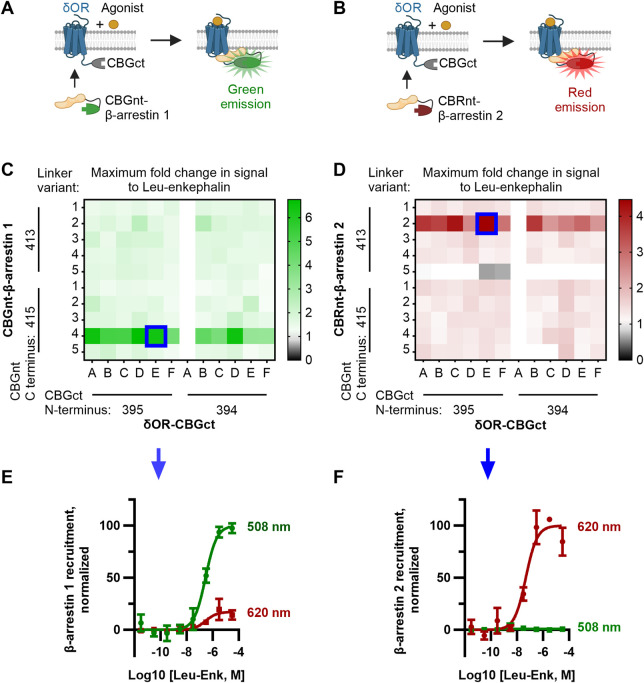
Optimization of CBGnt-β-arrestin 1 and CBRnt-β-arrestin 2 yields re-complemented enzymes with spectrally distinct readouts for β-arrestin 1 and β-arrestin 2 recruitment to a common δOR-CBGct construct. **(A, B)** Schematics showing assay design whereby either CBGnt-β-arrestin 1 **(A)** or CBRnt-β-arrestin 2 **(B)** is recruited to the δOR that results in the refolding of the click beetle luciferase and generation of a bioluminescent signal. CBGnt and CBGct: N- and C-terminal fragments of click beetle green luciferase, respectively. CBRnt: N-terminal fragment of click beetle red luciferase. **(C, D)** Maximum fold response to the endogenous δOR ligand leu-enkephalin for β-arrestin 1 **(C)** and β-arrestin 2 **(D)** constructs with various linkers and luciferase fragment termini. Optimal constructs with a common δOR-CBGct construct highlighted by blue squares. The linker sequences tested for δOR-linker-CBGct are A, GS; B, GS (SGGGG); C, GS (SGGGG)_2_; D, GS (SGGGG)_3_; E, GS (SGGGG)_4_; and F, GS (SGGGG)_5_. Linker sequences tested for CBGnt-linker-β-arrestin 1 and CBRnt-linker-β-arrestin 2 are 1, G (GGGGS); 2, S (GGGGS)_2_; 3, S (GGGGS)_3_; 4, S (GGGGS)_4_; and 5, S (GGGGS)_5_. Construct pairings showing zero overall response change could often not be reliably fit and are so displayed as having a fold change of top/bottom = 1. **(E)** Normalized emission showing that red (620 nm) emission is minimal when CBGct-β-arrestin 1 is recruited to δOR-CBGct. **(F)** Conversely, almost no green (508 nm) light is emitted when CBRnt-β-arrestin 2 is recruited to δOR-CBGct, which indicates independent signals for each β-arrestin.

To mutate the original constructs and generate the linker library, we used the NEBuilder HiFi DNA Assembly kit (New England Biolabs). We designed primers encoding the desired linkers and flanking regions that overlap the sequences of the δOR, arrestins, and luciferase fragments according to the manufacturer’s guidelines. Two sets of primers were ordered for each construct to test different termini of the luciferase fragments. For δOR-CBGct, the primers were ordered to include the fragments CBG (395–542) and CBG (394–542). For CBGnt-β-arrestin 1, CBG (1–413) and CBG (1–415) were tested. For CBRnt-β-arrestin 2, CBR (1–413) and CBR (1–415) were tested. Six linkers were tested for δOR-CBGct and five for CBGnt-β-arrestin 1/CBRnt-β-arrestin 2, which are shown in Figure 1 and Supplementary Figure S1.

### 2.2 ClickArr assay

#### 2.2.1 General protocol

A detailed protocol is included in the Supplementary Methods. Briefly, HEK293 cells were transfected using the X-tremeGENE 9 reagent (Roche) using an equimolar ratio of each construct. For the screen, carrier DNA was substituted for the DNA of the second β-arrestin construct so that all the transfections had the same total DNA. After 2 days, the transfected cells were rinsed with DPBS (Gibco), dissociated with trypsin-EDTA (Gibco), and 15,000–22,000 cells seeded into wells in a 384-well plate in Opti-MEM (Gibco). The plates were sealed with AeraSeal (MilliporeSigma) and equilibrated in a 37°C, 5% CO_2_ incubator for 30 min. A 2 mM solution of D-luciferin (GoldBio) was prepared in assay buffer (AB, HBSS (Gibco) + 20 mM HEPES). After equilibration, 7.5 μL of this was added to each well, and the plate was spun, resealed, and returned to the incubator. Agonist solutions were prepared at 4× concentration in AB, and 5 μL was added to the wells after 30 min. The plates were again spun, resealed, and returned to the incubator. A BioTek Synergy 4 plate reader equipped with 508/20 and 620/10 EM filters was preheated to 37°C, and readings performed 30 min after drug addition (0.5-s integration time).

#### 2.2.2 Kinetic experiments with ClickArr

In our hands, 20 wells could be read in both colors with a time step of 30 s. Thus, we ran each kinetic assay replicate with the indicated concentrations of agonists (four concentrations of leu-enkephalin and TAN67) in duplicate, along with two vehicle wells to control for signal drift.

### 2.3 PathHunter assays

PathHunter assays were run as previously described (see Supplementary Methods) (Chiang et al., 2016).

### 2.4 Data analysis

The Synergy 4 plate reader was controlled using Gen5 v2.04 software. The data were analyzed and plotted using the GraphPad Prism 9 software. Dose–response curves were analyzed using the three-parameter “log (agonist) vs. response” algorithm in Prism 9. For the construct screen, each pair of either δOR-CBGct × CBGnt-β-arrestin 1 or δOR-CBGct × CBRnt-β-arrestin 2 was run in a single dose–response curve in duplicate. The fold change data in the screen were calculated as the top/bottom of each fit. The optimal trio was selected by maximizing the signal change with the constraint that both β-arrestin isoform constructs had to pair with a common δOR construct. In figures with the full ClickArr assay, a replicate is an independent transfection/plate, with each point run in duplicate. In endpoint assays with multiple agonists, plates always included a dose–response curve with leu-enkephalin. To control for differences in isoform expression, data for each agonist at each isoform were normalized to its own baseline and the change in the signal was scaled so that the change in the response to leu-enkephalin was 100%. Efficacy bias is therefore calculated as the difference in the relative efficacy of a given agonist for β-arrestin 1 minus its relative efficacy for β-arrestin 2 (French et al., 2022). In order to generate a more authentic run error, the data given in Supplementary Tables S1, S2 represent the means and error of the fit values from the independent assays rather than the fit error on the averaged curves in the main text. All t-tests were two-tailed.

In kinetic experiments, the signal for each well was normalized to its mean baseline over the 2 min preceding the drug addition, and the mean normalized signal from the vehicle wells was then subtracted to control for drift. We found that a 5-min baseline is sufficient to equilibrate to the plate reader. As for the endpoint assays, data for both β-arrestin isoforms were acquired concomitantly on the same plate. Additional details on the kinetic fits of the β-arrestin dynamic data can be found in the Supplementary Methods.

## 3 Results

### 3.1 Development of the ClickArr assay

Our design for ClickArr takes advantage of the bright click beetle green (CBG) and click beetle red (CBR) luciferases, both of which catalyze the oxidation of the same substrate, D-luciferin, but emit light at different wavelengths (Wood et al., 1989; Almond et al., 2003). It was previously demonstrated that the N-terminal fragment of both CBG and CBR can reversibly complement a common C-terminal fragment from CBG and retain the spectral properties of full-length proteins (Villalobos et al., 2010). We reasoned that fusing a C-terminal fragment of CBG (CBGct) to a GPCR and then fusing N-terminal fragments of CBG (CBGnt) and CBR (CBRnt) to β-arrestin 1 and β-arrestin 2, respectively, would yield spectrally distinct signals for the recruitment of either isoform. For the GPCR construct, we fused CBGct to the C-terminus of the δOR. The δOR is an apt choice for our proof-of-concept as 1) there is a previously established divergence of roles for β-arrestin 1 and 2 at this receptor (Pradhan et al., 2016); 2) there are readily available agonists that range from partial recruiters to super-recruiters of β-arrestins relative to the endogenous agonist leu-enkephalin to serve as test compounds (Chiang et al., 2016; Blaine et al., 2022); and 3) the δOR is a promising drug target for a range of neurological disorders (Pradhan et al., 2011). As a class A receptor, we expected the δOR to interact less with β-arrestin 1 than with β-arrestin 2 (Qiu et al., 2007). Thus, we fused the brighter CBGnt to the N-terminus of β-arrestin 1 and the dimmer CBRnt to the N-terminus of β-arrestin 2 to help balance the signal-to-noise ratio for recruitment of each β-arrestin isoform (Figures 1A,B, Supplementary Figure S1) (Miloud et al., 2007).

We adopted a linker library previously used for optimizing split click beetle luciferase complementation assays (Misawa et al., 2010) to generate a small library of δOR and β-arrestin constructs fused to fragments of click beetle luciferase (see the Methods section, Supplementary Figure S1). In addition to varying the linker, we evaluated two terminal residues for (i.e., the length of) each click beetle luciferase fragment used. Since we optimized the ClickArr assay to run in 384-well format, we could generate a full dose–response curve for each pair of constructs in our screen. Plotting the maximum fold change, top/bottom, in the signal following the addition of saturating leu-enkephalin revealed that most combinations produced at least a 1.5-fold increase in the signal for β-arrestin 1 and 1.3-fold for β-arrestin 2, likely due to the flexible nature of the linkers used (Figures 1C,D). The exception is CBRnt^1-413^-S(GGGGS)_5_-β-arrestin 2, which produced no response with any δOR construct. Interestingly, for each β-arrestin isoform, there was a critical linker/fragment terminus combination that provided a much higher response than the others: CBGnt^1-415^-S(GGGGS)_4_-β-arrestin 1 and CBRnt^1-413^-S(GGGGS)_2_-β-arrestin 2. In contrast, the signal change was less dependent on the δOR fusion construct used, with the exception of δOR-CBGct constructs containing CBGct^394-542^ and a GS linker (Linker A, Figures 1C,D), which showed no response. This was our shortest linker examined, and so it seems this combined with the shorter CBGct^394-542^ fragment prevented the fragments from reaching a proper positioning for refolding. Overall, our β-arrestin isoform screens yielded hits with >400% signal change at a common δOR-CBGct construct. Critically, these top hits for CBGct-β-arrestin 1 and CBRnt-β-arrestin 2 maintained their spectrally distinct emission windows for 508 nm and 620 nm emission channels, respectively (Figures 1E,F).

### 3.2 ClickArr assay shows β-arrestin isoform bias at δOR

**FIGURE 2:**
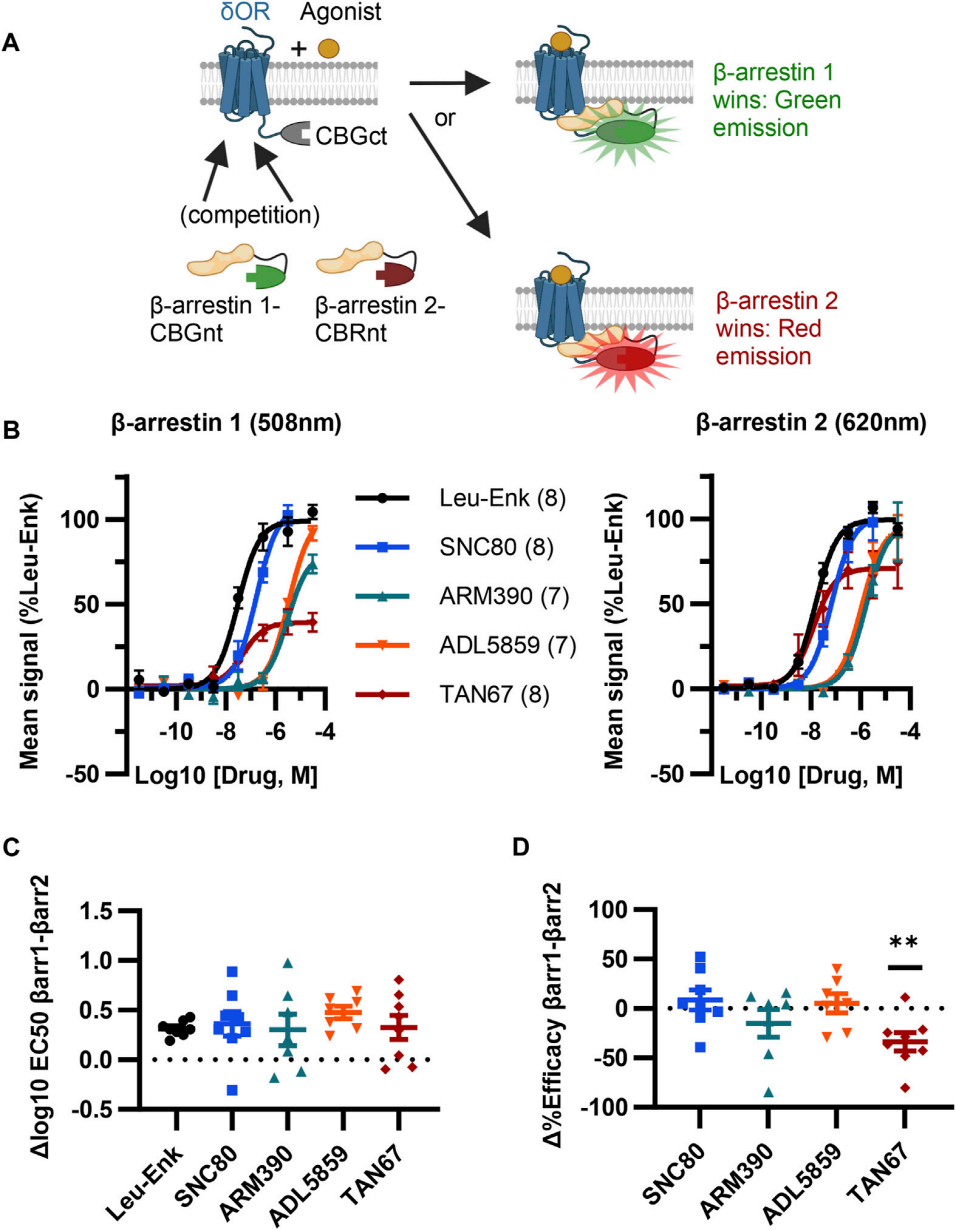
ClickArr screen demonstrates β-arrestin isoform bias at the δOR. **(A)** Cartoon showing ClickArr assay allows a spectrally distinct readout of β-arrestin isoform competition for δOR receptors in the same cell. **(B)** ClickArr assay results using top screen hits from Figures 1C, D that show recruitment for β-arrestin 1 (left) and β-arrestin 2 (right). All three constructs were co-transfected into cells at an equimolar ratio and 2 days later seeded into 384 well plates before being challenged with different δOR agonists, as described in the Methods section. Legend: agonist (n). **(C)** Differences in log10 EC_50_ values (Δlog10 EC_50_, β-arrestin 1 − β-arrestin 2) from fitted normalized data in **(B)**. No significant differences in Δlog10 EC_50_ are seen between test agonists and the reference agonist—leu-enkephalin (Leu-Enk). **(D)** However, TAN67 shows significantly lower percent efficacy (normalized to leu-enkephalin) for β-arrestin 1 than for β-arrestin 2. **, *p* < 0.01 relative to Δ%efficacy for leu-enkephalin (defined as zero). Plotted as mean ± SEM.

Given our screen results, δOR-GS(SGGGG)_4_-CBGct^395-542^, CBGnt^1-415^-S(GGGGS)_4_-β-arrestin 1, and CBRnt^1-413^-S(GGGGS)_2_-β-arrestin 2 were carried forward as the “ClickArr” assay (Figure 2A). Class A GPCRs such as the δOR recruit β-arrestin 2 more strongly than β-arrestin 1, demonstrating that some receptors can distinguish between β-arrestin isoforms (Oakley et al., 2000). This raised to us the possibility that different receptor–agonist complexes could have a relative bias in β-arrestin isoform recruitment. To establish whether ClickArr could detect differences in β-arrestin isoform recruitment, we characterized our reference compound leu-enkephalin in parallel with a small panel of tool agonists (Figure 2B; Supplementary Table S1). The agonist rank order in potency for β-arrestin 1 and 2 recruitment was similar (Figure 2C). However, we found a significant shift in the efficacy values for TAN67 relative to the endogenous agonist leu-enkephalin [one sample t-test, (t, df) = (3.663, 7), *p* = 0.008], with TAN67 recruiting β-arrestin 2 with higher relative efficacy than β-arrestin 1 (Figure 2D). This result confirms our hypothesis that GPCRs can bias recruitment between β-arrestin isoforms depending on the agonist bound.

### 3.3 Comparison to δOR PathHunter assays

**FIGURE 3:**
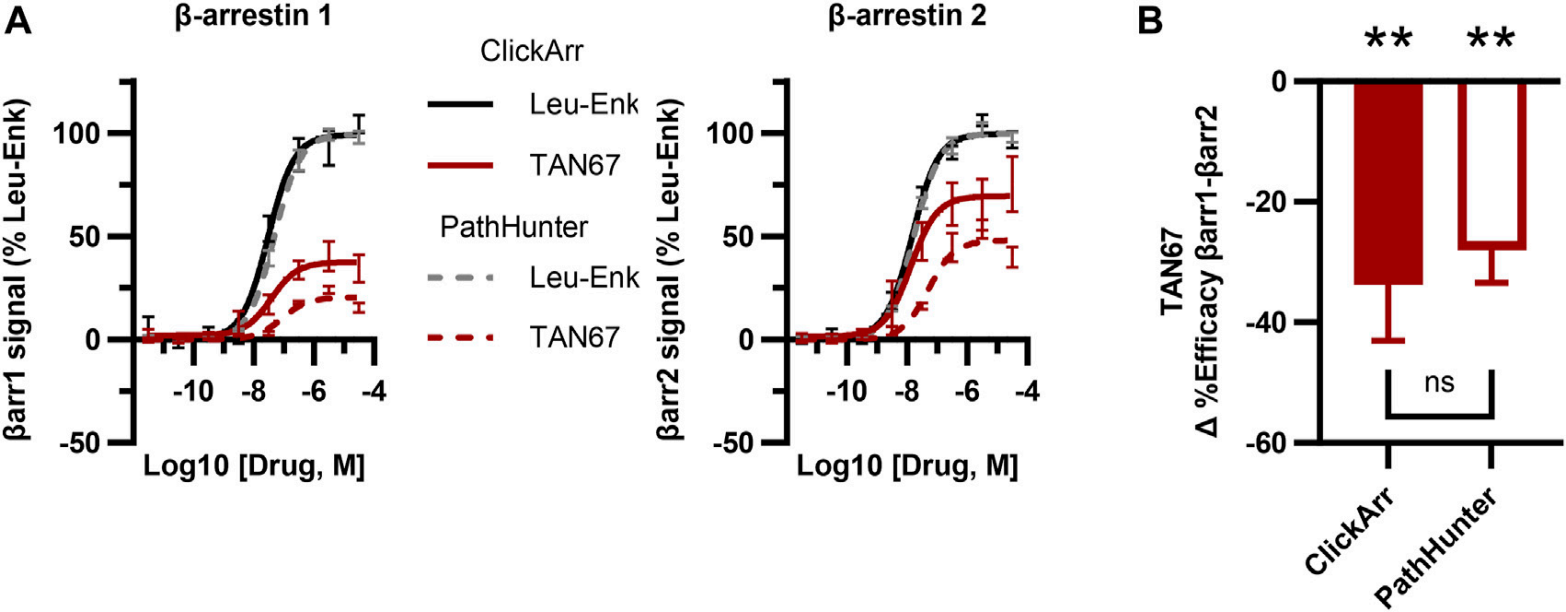
The ClickArr assay compares well to a commercial assay for β-arrestin recruitment to the δOR. **(A)** ClickArr curves for leu-enkephalin (Leu-Enk) and TAN67 compare well to the PathHunter assay, the state-of-the-art assay for β-arrestin recruitment. **(B)** The PathHunter assay confirms the TAN67 efficacy bias detected by the ClickArr assay. **, *p* < 0.01 with respect to Δ%efficacy for leu-enkephalin (defined as zero). ClickArr, n = 8; PathHunter, n = 5. Plotted as the mean ± SEM.

The PathHunter assay is a commercial assay used for the high-throughput evaluation of the recruitment of a single β-arrestin isoform to a receptor and is a standard bearer in this field (Spillmann et al., 2020). To compare the performance of ClickArr to that of the PathHunter assay and independently confirm the presence of β-arrestin isoform bias at the δOR, we ran the δOR:β-arrestin 1 and δOR:β-arrestin 2 PathHunter assays. We found that the performance of leu-enkephalin is nearly identical in the ClickArr and PathHunter assays (Figure 3A; Supplementary Figure S2; Supplementary Tables S1, S2). The ClickArr assay is slightly more sensitive than the PathHunter assay to the partial agonist TAN67, although both assays report TAN67 as a partial agonist (Figure 3A; Supplementary Figure S2A,B; Supplementary Tables S1, S2). Similar to ClickArr, the PathHunter assay did not show an isoform-specific shift in relative potency for TAN67 when compared to the reference agonist leu-enkephalin (Supplementary Figure S2C). Importantly, the PathHunter assay confirmed the presence of an efficacy bias of TAN67 for β-arrestin 2 recruitment over β-arrestin 1 [one sample t-test, (t, df) = (5.162, 4), *p* = 0.0067] (Figure 3B). Therefore, our results with the PathHunter assay validate the β-arrestin isoform bias detected in the ClickArr assay and support the idea that GPCR–agonist complexes can distinguish between β-arrestin isoforms.

### 3.4 Measuring β-arrestin dynamics with ClickArr

**FIGURE 4:**
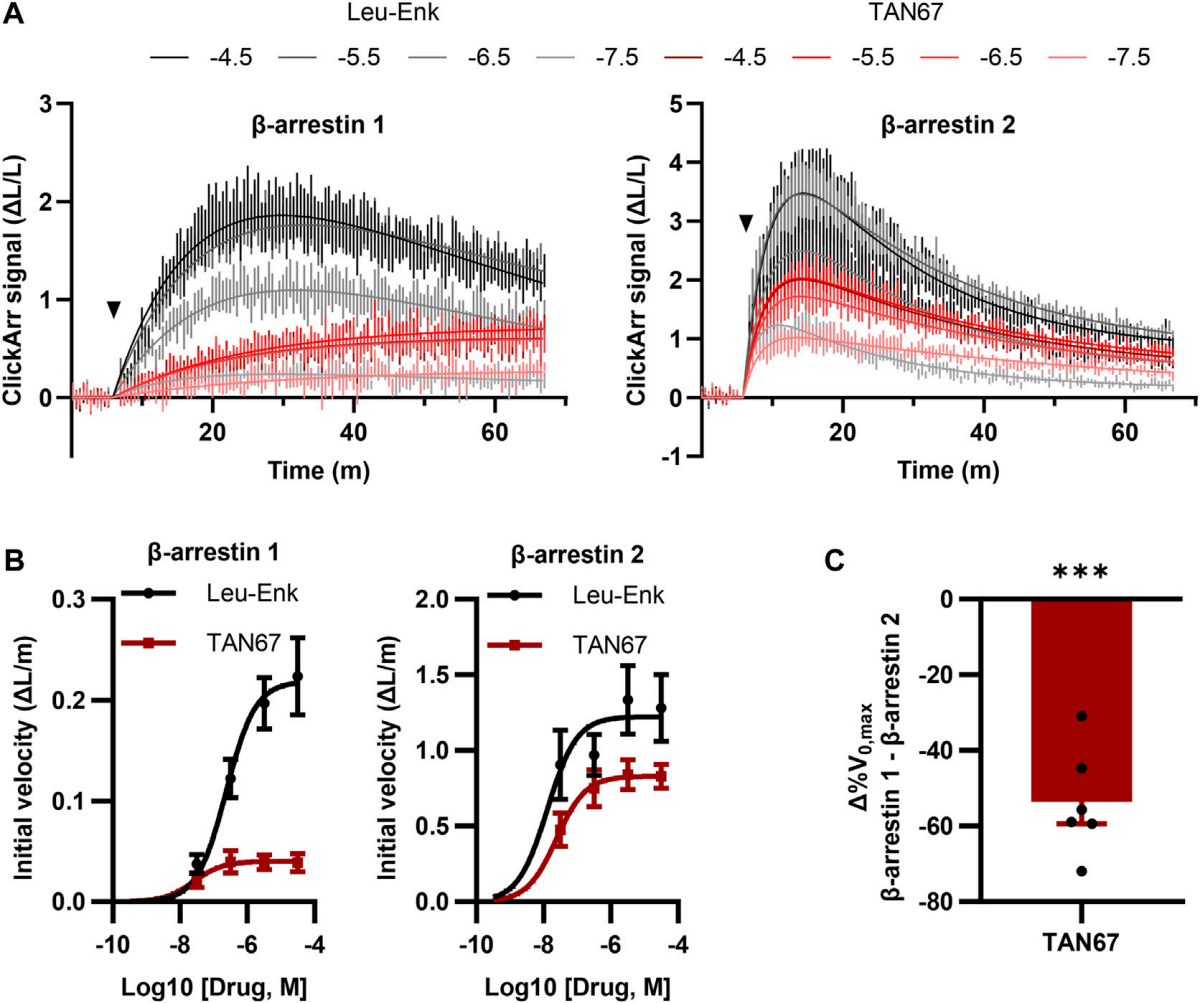
ClickArr reports arrestin dynamics in 384-well plate format, and TAN67 β-arrestin isoform bias is confirmed by reaction velocities. **(A)** Following a 5-minute equilibration, either leu-enkephalin or TAN67 was added to wells (triangle) and the evolution in the signal for β-arrestin 1 and 2 recorded over time. The legend is log10 (concentration, M). **(B)** Plotting the initial velocity vs concentration for each drug and β-arrestin reveals β-arrestin isoform–specific differences in TAN67 dynamics relative to leu-enkephalin. **(C)** The difference in the relative maximum initial velocity (%V_0,max_) between β-arrestin 1 and 2 confirms a bias toward β-arrestin 2 over β-arrestin 1 for TAN67 at the δOR. ***, *p* < 0.001 relative to Δ%V_0,max_ for leu-enkephalin (defined as zero). All graphs, n = 6. Plotted as mean ± SEM.

A unique feature of ClickArr over endpoint assays such as PathHunter is that ClickArr is a live-cell assay, which should enable kinetic studies of β-arrestin recruitment. Kinetic drug screening offers higher information content than endpoint screening and can yield mechanistic insights into how a molecule interacts with a signaling pathway (Hoare et al., 2022). Importantly, kinetic drug screening can also be a more sensitive and reliable measure of bias than endpoint screening (Hoare et al., 2020a). To demonstrate the use of ClickArr as a kinetic screen, we recorded β-arrestin 1 and 2 recruitment to δORs in the presence of leu-enkephalin or TAN67 for over 1 h (Figure 4A). Plotting the initial reaction velocity against the concentration shows that ClickArr reports a bias for TAN67 toward β-arrestin 2 recruitment [one sample t-test, (t, df) = (9.295, 5), *p* = 0.0002] relative to leu-enkephalin (Figures 4B,C). Thus, ClickArr consistently reports a β-arrestin isoform bias toward β-arrestin 2 for TAN67 relative to leu-enkephalin whether it is used as an endpoint assay or a kinetic assay.

## 4 Discussion

Herein, we presented data validating ClickArr as a novel assay architecture that simultaneously reports the recruitment of both β-arrestin isoforms as they compete for binding to a GPCR, using the δOR as the proof of concept. We found that certain agonists complexed with the δOR can have different relative efficacies for recruiting the two β-arrestin isoforms (Figure 2D).

We based our design for ClickArr on the complementation of click beetle luciferases with different emission spectra to a common luciferase fragment fused to the δOR. To optimize our design, we screened each CBGnt-β-arrestin 1 and CBRnt-β-arrestin 2 construct against the δOR-CBGct constructs, varying the linkers and termini of each fragment. Unlike in a similar screen involving the somatostatin receptor 2 (SSTR2), we found a more discrete effect of linker–terminus combinations when recruiting to the δOR in that two β-arrestin constructs had exceptional signal change. As every construct was evaluated on at least two plates, ruling out plate/time artifacts, it is not immediately clear why the linker–fragment terminus combinations in CBGnt^1-415^-S(GGGGS)_4_–β-arrestin 1 and CBRnt^1-413^-S(GGGGS)_2_–β-arrestin 2 had such strong effects. Interestingly, Misawa et al. (2010) optimized their choice of fragment termini using FRB/FKBP binding and found that, generally, CBGct^394-542^ substantially outperformed CBGct^395-542^. However, when we performed our optimization on the δOR, we found that, in fact, CBGct^395-542^ performed better, although the two were comparable. This could be due to the different orientations of the fragments in the δOR:β-arrestin complex compared to the FRB/FKBP complex. In any case, we found constructs yielding greater than 400% signal change for both β-arrestin isoforms using a limited screen of only 10 constructs per isoform.

To create ClickArr, we took advantage of the ability of N-terminal CBG and CBR luciferase fragments fused to β-arrestin isoforms 1 and 2 to complement a common CBG C-terminal fragment fused to the δOR. The primary advantages of this approach are that ClickArr reports the recruitment of both β-arrestin isoforms simultaneously as they compete for δOR in the same cell and that this complementation is reversible, enabling both high-throughput endpoint and kinetic assay modes. Moreover, as CBG and CBR both utilize D-luciferin as their substrate, only one luciferin needs to be added to the cell. Other split-luciferase systems such as the NanoLuc-based NanoBiT system are also reversible and can even report recruitment without receptor modification (Janetzko et al., 2022) but can only report on a single β-arrestin isoform in a cell. Similarly, the split-galactosidase PathHunter assay requires a unique cell line for each β-arrestin isoform and cannot report dynamics.

Alternative β-arrestin recruitment architectures also include enzyme-induced cleavage of transcription factors and bioluminescent resonance energy transfer (BRET)-based assays (Kroning and Wang, 2022). For example, the cleavage-dependent Tango assay system was recently extended to include β-arrestin 1 (Zeghal et al., 2023). However, the Tango system requires unique cell lines for each isoform and cannot report β-arrestin dynamics. Since the cleaved transcription factor is fused to the receptor, it is unclear how this could be redesigned to generate independent signals for each β-arrestin isoform in the same cells. In contrast, BRET-based assays are generally capable of reporting effector dynamics (Salahpour et al., 2012). As an added benefit, BRET approaches can forgo direct modification of the GPCR, although they still generally involve modifying the effector proteins and highly expressing a lipid-tethered bioluminescent or fluorescent protein. Conceivably, BRET approaches could be adapted for the multiplexed reporting in ClickArr by tethering a blue donor molecule to the membrane/GPCR, and fusing green and red acceptors to β-arrestin 1 and 2, respectively. One challenge of this approach would be that in resonance energy-transfer mechanisms such as BRET, the spatial requirements for efficient energy transfer are dependent on the spectral properties of each donor and acceptor (De et al., 2013). As a result, the blue-green and blue-red BRET pairs would reach maximum efficiency at different separation lengths. Thus, more optimization might be needed in the development of such a screen. Furthermore, since β-arrestins can have different orientations on different GPCRs, significant re-tuning of the β-arrestin constructs could be required for each GPCR. In contrast, the relative indifference of the ClickArr assay to the choice of linker on the δOR (Figure 1) suggests that there is a high degree of tolerability in this architecture for orientation and spacing of the luciferase fragments. Thus, while there are advantages and disadvantages to each approach, in particular that the BRET and Tango architectures are already available for a wide range of GPCRs (Avet et al., 2022; Zeghal et al., 2023), the ClickArr architecture may be the optimal choice for researchers interested in evaluating β-arrestin isoform dynamics and/or β-arrestin isoform bias in the same cells. Regardless of the modality chosen, our results demonstrating β-arrestin isoform signaling bias should help motivate researchers to evaluate new compound signaling profiles at both β-arrestin isoforms.

Our ClickArr assay identified a β-arrestin isoform bias for TAN67 at the δOR relative to the endogenous δOR agonist leu-enkephalin (Figure 2). These data support previous work on the C-C chemokine ligand 5 (CCL5) analog compound 5P14-RANTES, which has a stronger efficacy for recruiting β-arrestin 2 than it does for recruiting β-arrestin 1 to the C-C chemokine receptor 5 (CCR5) relative to the CCR5 endogenous agonist CCL5 (Martins et al., 2020). In the previous work, the authors used two separate cell lines to measure β-arrestin 1 and β-arrestin 2 recruitment. Therefore, our results extend this work by suggesting that this β-arrestin isoform bias can occur when β-arrestin isoforms compete for receptors in the same cell. Together, these results suggest the possibility that β-arrestin isoform bias is a common ability of GPCRs. Conceivably, ClickArr assays could be developed for GPCRs beyond the δOR, which would extend ClickArr to a diverse array of prospective drug targets. Based on our results, we suggest researchers seeking to extend the ClickArr assay clone 5–10 CBGct fusions to the C-terminus of their GPCR of choice and then screen these against the β-arrestin constructs developed here.

Our identification of TAN67 as a β-arrestin 2-preferring δOR agonist highlights how ClickArr could help identify drugs with β-arrestin isoform bias that have reduced off-target pathway activation and subsequently improved side-effect profiles. For example, at the δOR, β-arrestin 2 recruitment is associated with necessary receptor internalization and resensitization, whereas β-arrestin 1 recruitment targets δORs for degradation leading to rapid tachyphylaxis (Pradhan et al., 2016). As δORs are a target for chronic indications, such as migraine, neuropathic pain, and alcohol use disorder, avoiding β-arrestin 1–induced tachyphylaxis would improve the repeated performance of therapeutics at this receptor (Meqbil and van Rijn, 2022). In this vein, TAN67 has preclinical efficacy in reducing alcohol intake in mice (Chiang et al., 2016); thus our finding that arrestin recruitment at TAN67 is biased away from β-arrestin 1 could make this a more exciting lead candidate.

As a live-cell assay that utilizes the reversible folding/unfolding of click beetle luciferase enzymes, ClickArr is usable as a readout for β-arrestin dynamics (Figure 4). Although a detailed analysis of receptor trafficking is outside the scope of this work, we can make some early comparisons to published literature. We first note that the best-fit kinetic equation for leu-enkephalin–induced recruitment of β-arrestin 1 comes from a “rise-and-fall to baseline” model for a case where the receptor is desensitized and degraded (Hoare et al., 2020b) (also see Supplementary Methods), which is consistent with the role of β-arrestin 1 previously derived from a combination of *in vitro* BRET and *in vivo* knockout experiments looking at the high-internalizing agonist SNC80 (Pradhan et al., 2016). By contrast, our leu-enkephalin β-arrestin 2 data were a best fit to a “rise-and-fall to steady state” model, which can occur through several combinations of mechanisms. These include resensitization, which would be consistent with the role of β-arrestin 2 recruited to δOR by SNC80 derived by Pradhan et al., as well as signaling at internalized receptors. TAN67 displayed a pattern different from that of either the high-internalizing or low-internalizing agonists investigated by Pradhan et al. Our kinetic models (see Supplementary Methods) suggest that the TAN67-δOR engagement of β-arrestin 2 follows a similar mechanism to leu-enkephalin and possibly SNC80, but its engagement with β-arrestin 1 suggests that it drives persistent association that is deficient in either receptor desensitization or degradation compared to leu-enkephalin. Identifying the exact nature of these interactions requires further investigation, but these early results from ClickArr support the use of high-throughput kinetic screens in parsing the nature of how the agonist:receptor complexes interact with effector systems (Hoare et al., 2020a).

Although construct expression levels were not measured, both isoforms generate a robust signal that performs on par with the PathHunter assay (Figure 3, Supplementary Figure S2). Furthermore, bias is considered with respect to leu-enkephalin, and this normalization should control for differences in signals stemming from differences in isoform expression, which varies *in vivo* (Gurevich et al., 2002). Another potential limitation of this study is that the observed β-arrestin isoform bias for TAN67 is partial, that is, recruitment of neither isoform is completely eliminated. Thus, the full extent to which different receptors can be biased toward a given isoform is not yet known and is likely to vary between receptors. It is also not yet known whether β-arrestin isoform bias can be strengthened to a clinically meaningful degree. Nonetheless, the ClickArr architecture has opened a new dimension of agonist bias to explore at GPCRs and is itself an effective, high-throughput tool for investigating these questions.

## Data Availability

The raw data supporting the conclusion of this article will be made available by the authors, without undue reservation.

## Supplementary material

Supplementary Table S1 Fit values for δOR ClickArr assay.

Supplementary Table S2 Fit values for PathHunter assay.

Supplementary Figure S1 Orientations of fragments and linker sequences used in screen. δOR, δ opioid receptor; G, glycine; S, serine; CBGct, C-terminal fragment of click beetle green luciferase; CBGnt, N-terminal fragment of click beetle green luciferase; CBRnt, N-terminal fragment of click beetle red luciferase. See methods for additional information on fragment lengths and vector construction.

Supplementary Figure S2 TAN67 potency, efficacy, and potency relative to leu-enkephalin between ClickArr and PathHunter assays at the δOR. **(A)** TAN67 Log10(EC50) values for β-arrestin 1 (left) and β-arrestin 2 (right) for ClickArr and PathHunter assays. Data normalization by Y = y^−2^ and subsequent ANOVA shows a significant difference between assays (F(1, 22) = 5.673, *p* = 0.0263). However, a post-hoc Sidak test did not reveal a significant difference between tests for either isoform. **(B)** TAN67 efficacy normalized to leu-enkephalin for the ClickArr and PathHunter assays. Data normalization by Y = sqrt(y) and subsequent ANOVA show a significant difference between tests (F(1, 22) = 5,496, *p* = 0.0285) and isoform (F(1, 22) = 15.79, *p* = 0.0006). However, a post-hoc Sidak test did not reveal a significant difference between tests for either isoform. **(C)** Similar differences in potency (Log10 EC50) β-arrestin 1 - β-arrestin 2 are seen between the ClickArr and PathHunter assays for both leu-enkephalin and TAN67. No significant isoform-specific effects on TAN67 potency relative to leu-enkephalin were reported by either assay. **(D)** Raw values for the baseline and peak fit values for the leu-enkephalin dose-response curves for the ClickArr (left), β-arrestin 1 PathHunter (middle), and β-arrestin 2 PathHunter (right). All graphs mean ± SEM. n = 8, ClickArr; 5, PathHunter.
