# Supplementary material for: ClickArr: a novel, high-throughput assay for evaluating β-arrestin isoform recruitment
Source: Front Pharmacol. 2023 Nov 7;14:1295518. doi: 10.3389/fphar.2023.1295518 (PMC10662323; doi:10.3389/fphar.2023.1295518)
Supplement: Supplementary file 1 [file DataSheet1.PDF]

Supplementary Material

1 Supplementary Tables

Supplementary Table S1: Fit values for δOR ClickArr assay

	β-arrestin 1			β-arrestin 2		
	pEC_{50}	EC_{50} (nM)	E_{max} (%)	pEC_{50}	EC_{50} (nM)	E_{max} (%)
Leu-enkephalin	7.49 (0.11)	32.2	100	7.81 (0.11)	15.7	100
SNC80	6.81 (0.17)	153.5	110 (7.3)	7.15 (0.08)	71.4	102 (11)
ARM390	5.53 (0.11)	2978	82.4 (6.5)	5.83 (0.08)	1487	97.6 (19)
ADL5859	5.47 (0.05)	3401	102 (4.7)	5.95 (0.09)	1134	97.1 (13)
TAN67	7.54 (0.29)	28.7	37.2 (5.2)	7.90 (0.19)	12.6	71.6 (12)

All values mean (SEM); pEC_{50} values reported in -log10(M). n = 7-8.

Supplementary Table S2: Fit values for PathHunter assay

	β-arrestin 1			β-arrestin 2		
	pEC_{50}	EC_{50} (nM)	E_{max} (%)	pEC_{50}	EC_{50} (nM)	E_{max} (%)
Leu-enkephalin	7.30 (0.07)	50.3	100	7.77 (0.05)	17.0	100
TAN67	7.08 (0.07)	83.9	20.4 (1.4)	7.30 (0.04)	51.9	48.4 (5.2)

All values mean (SEM); pEC_{50} values reported in -log10(M). n = 5.

2 Supplementary Figures and Tables

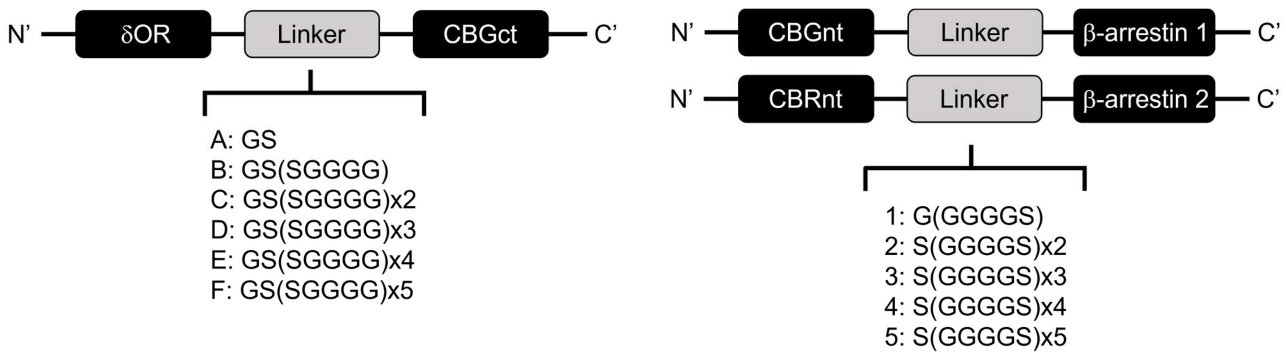

Supplementary Figure S1: Orientations of fragments and linker sequences used in screen. δOR, δ opioid receptor; G, glycine; S, serine; CBGct, C-terminal fragment of click beetle green luciferase; CBGnt, N-terminal fragment of click beetle green luciferase; CBRnt, N-terminal fragment of click beetle red luciferase. See methods for additional information on fragment lengths and vector construction.

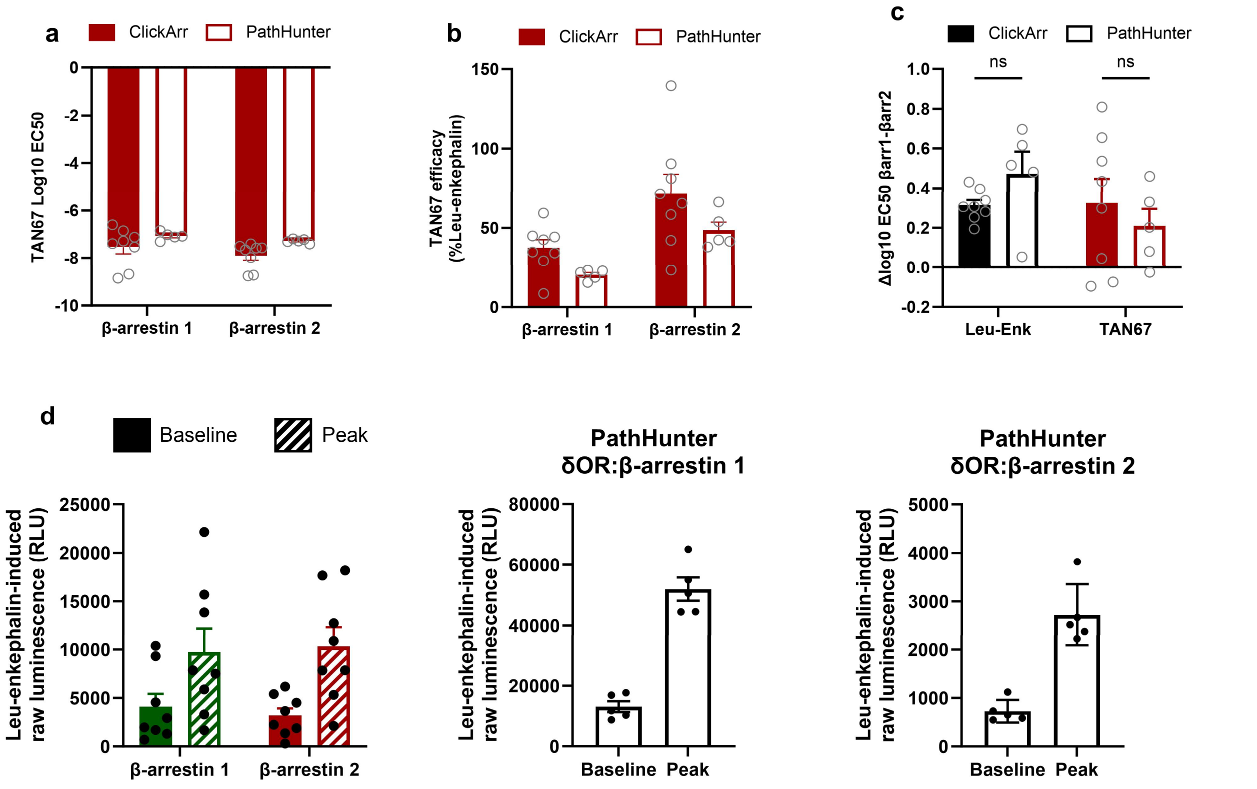

Supplementary Figure S2: TAN67 potency, efficacy, and potency relative to leu-enkephalin between ClickArr and PathHunter assays at the δOR. **(A)** TAN67 Log10(EC50) values for β-arrestin 1 (left) and β-arrestin 2 (right) for ClickArr and PathHunter assays. Data normalization by $Y = y^{-2}$ and subsequent ANOVA shows a significant difference between assays ($F(1, 22) = 5.673$, $p = 0.0263$). However, a post-hoc Sidak test did not reveal a significant difference between tests for either isoform. **(B)** TAN67 efficacy normalized to leu-enkephalin for the ClickArr and PathHunter assays. Data normalization by Y = sqrt(y) and subsequent ANOVA show a significant difference between tests ($F(1, 22) = 5{,}496$, $p = 0.0285$) and isoform ($F(1, 22) = 15.79$, $p = 0.0006$). However, a post-hoc Sidak test did not reveal a significant difference between tests for either isoform. **(C)** Similar differences in potency (Log10 EC50) β-arrestin 1 - β-arrestin 2 are seen between the ClickArr and PathHunter assays for both leu-enkephalin and TAN67. No significant isoform-specific effects on TAN67 potency relative to leu-enkephalin were reported by either assay. **(D)** Raw values for the baseline and peak fit values for the leu-enkephalin dose-response curves for the ClickArr (left), β-arrestin 1 PathHunter (middle), and β-arrestin 2 PathHunter (right). All graphs mean ± SEM. n = 8, ClickArr; 5, PathHunter.

3 Supplementary Methods

3.1 The PathHunter Assay

The PathHunter assays were carried out as previously described (French et al., 2022). U-2 osteosarcoma (U2OS)-hδOR-β-arrestin 1 PathHunter cells (RRID:CVCL_LA96, DiscoverX, Fremont, CA, USA) and Chinese hamster ovarian K1 (CHO-K1)-hδOR PathHunter β-arrestin 2 cells (RRID:CVCL_KY68, DiscoverX, Fremont, CA, USA) were each plated at 2,500 cells per 10 µl per well (one cell type per well) 1 day prior to stimulation with 2.5 µl drug solution for 90 minutes at 37C/5% CO2. Cells were then incubated with 6 µl cell PathHunter assay buffer (DiscoverX) for 60 minutes at room temperature per the manufacturer's protocol. Luminescence for each of these assays was measured using a FlexStation3 plate reader (Molecular Devices, Sunnyvale, CA, USA). For a given replicate, complete dose response curves were run for both cell types on the same plate and read together to reduce variability.

3.2 Additional analyses and statistical testing on dose-response curves

For added rigor, the extra sum-of-squares F test was applied to compare the peak efficacy (top parameter) and potency (log10EC50) of β-arrestin 1 and 2 for each agonist in Figures 2B and 3A. Because the curves are normalized to the top value of leu-enkephalin, this is an equivalent test for efficacy bias as we've calculated it in this manuscript. For log10EC50, it asks whether we can reject the hypothesis that the log10EC50 values are the same for both β-arrestin 1 and 2 for a given agonist. The results for the ClickArr top parameter are: SNC80, $F(1, 106) = 0.5326$, $p = 0.4671$; ARM390, $F(1, 106) = 2.457$, $p = 0.1200$; ADL5859, $F(1, 106) = 0.6349$, $p = 0.4273$; TAN67, $F(1, 121) = 16.78$, $p < 0.0001$. The results for the ClickArr log10EC50 value are: leu-enkephalin, $F(1, 122) = 5.231$, $p = 0.0239$; SNC80, $F(1, 106) = 4.780$, $p = 0.0310$; ARM390, $F(1, 106) = 1.511$, $p = 0.2217$; ADL5859, $F(1, 106) = 10.81$, $p = 0.0014$; TAN67, $F(1, 121) = 1.345$, $p = 0.2484$. The results for the PathHunter assay top parameter are: TAN67, $F(1, 74) = 56.31$, $p < 0.0001$. The results for the PathHunter Log10EC50 value are: leu-enkephalin, $F(1, 74) = 47.95$, $p < 0.0001$; TAN67, $F(1, 74) = 0.7264$, $p = 0.3968$.

Many studies evaluating signaling bias compute a single bias factor, $10^{\Delta\Delta\log(\tau/KA)}$, that attempts to consolidate the net effects of both the relative efficacy and potency of two agonists on two signaling pathways (Kenakin, 2019). For compatibility with these other works, we have computed the $\Delta\Delta\log(\tau/KA)$ ($\Delta\log(\tau/KA)_{barr1}$ - $\Delta\log(\tau/KA)_{barr2}$) for our agonists in the ClickArr assay, mean (95% CI): SNC80, 0.16 (-0.21, 0.53); ARM390, -0.11 (-0.50, 0.27); ADL5859, 0.00 (-0.38, 0.39); TAN67, -0.41 (-0.78, -0.03). To compute the $\Delta\Delta\log(\tau/KA)$ for each replicate, the dose-response curves were fit to the re-parameterized Black & Leff operational model in (Westhuizen et al., 2014), and the 95% confidence intervals estimated using the equations in (Kenakin et al., 2012).

3.3 Kinetic fits in Figure 4A

As expected for drugs of different bias at the δOR, the leu-enkephalin and TAN67 showed different kinetic profiles at β-arrestin 1 and 2. At β-arrestin 1, the responses to leu-enkephalin showed a biphasic response that were well fit to equation 3, "rise and fall to baseline" equation of Hoare et al. (Hoare et al., 2020):

Eq. 1 $y = \frac{C}{K1-K2}(e^{-K1(X-X0)} - e^{-K2(X-X0)})$,

where C is a parameter is a parameter determining the rise in the signal; X0 is the time of drug addition; and K1 and K2 are the rate constants of the rising and declining phases, respectively. In contrast the β-arrestin 1 response to TAN67 showed only a single-phase response and was fit to the one-phase association equation,

Eq. 2 $y = SSR * (1 - e^{-K1(X-X0)})$,

Where SSR is the steady-state level of signal and the other terms defined as for Eq. 1. The kinetic profiles for β-arrestin 2 recruitment showed yet another pattern and the responses to both agonists were fit to equation 4, "rise and fall to steady state" of Hoare et al. (Hoare et al., 2020):

Eq. 3 $y = SSR * (1 - De^{-K1(X-X0)} + (D-1)e^{-K2(X-X0)})$,

where D is a parameter determining the rise in the signal, and the other terms are defined as for Eqs. 1,2. Eq. 1 and 3 fit the β-arrestin 1 leu-enkephalin responses nearly equally well, with Eq. 1 ultimately chosen for parsimony. These equations were derived as operant models for GPCR signaling responses (Hoare et al., 2018, 2020). Eq. 1 describes a case where the receptor is both desensitized and the response signal degrades. Eq. 2 commonly describes situations where either the receptor is desensitized, the response signal degrades, or depletion of unbound effector, but not more than one of these simultaneously. Lastly, Eq. 3 can arise when either the receptor is resensitized or signaling persists at internalized receptors (Hoare et al., 2020). The applicability of these models supports previous findings for β-arrestin recruitment to GPCRs (Hoare et al., 2020, 2022).

3.4 Analysis of reaction velocities

Reaction velocity data in Figure 4 was calculated using the derivative of the fitted curves in Figure 4A at the time of drug addition. These were plotted against agonist concentration and fit to a 3-parameter (Hill constant = 1) sigmoidal binding function in Prism9 (GraphPad) with the "bottom" parameter constrained to zero.

3.5 Supplementary references

French, A. R., Gutridge, A. M., Yuan, J., Royer, Q. H., & van Rijn, R. M. (2022). Sex- and β-arrestin-dependent effects of kappa opioid receptor-mediated ethanol consumption. *Pharmacology Biochemistry and Behavior*, *216*(March), 173377. https://doi.org/10.1016/j.pbb.2022.173377

Hoare, S. R. J., Pierre, N., Moya, A. G., & Larson, B. (2018). Kinetic operational models of agonism for G-protein-coupled receptors. *Journal of Theoretical Biology*, *446*, 168–204. https://doi.org/10.1016/j.jtbi.2018.02.014

Hoare, S. R. J., Tewson, P. H., Quinn, A. M., Hughes, T. E., & Bridge, L. J. (2020). Analyzing kinetic signaling data for G-protein-coupled receptors. *Scientific Reports*, *10*(1), 1–23. https://doi.org/10.1038/s41598-020-67844-3

Hoare, S. R. J., Tewson, P. H., Sachdev, S., Connor, M., Hughes, T. E., & Quinn, A. M. (2022). Quantifying the Kinetics of Signaling and Arrestin Recruitment by Nervous System G-Protein

Coupled Receptors. *Frontiers in Cellular Neuroscience*, *15*(January), 1–25. https://doi.org/10.3389/fncel.2021.814547

Kenakin, T. (2019). *Biased Receptor Signaling in Drug Discovery*. *April*, 267–315. https://doi.org/10.1124/pr.118.016790

Kenakin, T., Watson, C., Muniz-Medina, V., Christopoulos, A., & Novick, S. (2012). A simple method for quantifying functional selectivity and agonist bias. *ACS Chemical Neuroscience*, *3*(3), 193–203. https://doi.org/10.1021/cn200111m

Westhuizen, E. T. Van Der, Breton, B., Christopoulos, A., & Bouvier, M. (2014). *Quantification of Ligand Bias for Clinically Relevant b 2 -Adrenergic Receptor Ligands : Implications for Drug Taxonomy s*.

4 ClickArr protocol printout

Our optimized protocol is as follows:

On day 1, seed HEK293 cells in an appropriate plate in full growth media (EMEM + 10% FBS). We typically seed 5 x 10^5 cells/well in a standard 6 well plate. On day 2, transfect cells with appropriate transfection method. We use XtremeGene9 (Roche), using a 1:1:1 mass ratio of δOR-CBGct:CBGnt-β-arrestin 1:CBRnt-β-arrestin 2 DNA. For double transfection of δOR with a single arrestin construct, we use salmon sperm DNA (Invitrogen) to substitute for the construct being excluded. Transfections are optimized and carried out according to the manufacturer's guidelines.

On day 4, the following assay procedure is started 38-40 hours following transfection.

1. Rinse cells with DPBS and digest cells with trypsin-EDTA solution (Gibco)
2. Dissociate cells in 2x volume Opti-MEM (Gibco)
3. Pellet cells at 5.5 min/300xg and resuspend to 2-3x10^6/mL in Opti-MEM.
4. Seed 7.5 mL cell suspension/well of a 384 well plate.
5. Spin, cover with AeraSeal (Millipore-Sigma) to prevent drying out, and put in 37C incubator for 30 minutes.
6. Make up a 2mM luciferin solution using either D-Luciferin frozen stocks or powder in Assay buffer (AB)
    a. We find that aliquots of a 100 mM Na-luciferin stock made in assay buffer (AB, 980 mL HBSS (Gibco), 20 mL 1M HEPES (Gibco)) can be frozen at -80C and used for at least two weeks.
7. Take out plate and briefly spin to bring down any moisture condensing on the upper part of the plate.
8. Pipette 7.5 mL 2 mM luciferin mix in each well and briefly centrifuge. Recover and put back in 37C and incubate for 30 minutes.
    a. Prepare drug solutions in AB at 4 times the desired final concentration.
9. Take out plate and briefly centrifuge. Add 5 mL of drug solution to each well. Spin plate, reseal, and incubate for 30min at 37C.

Pre-heat plate reader to 37C and measure luminescence (0.5s integration time). This study used a Biotek Synergy4 plate reader with 508/20 and 620/10 EM filters.
